# The impact of depression on mortality among older adult patients with hypertension: a systematic review and meta-analysis

**DOI:** 10.3389/fpubh.2025.1603785

**Published:** 2025-07-31

**Authors:** Ze Fang, Tao Huang, Qiongfang Zhang, Lili Shi, Rui Huang

**Affiliations:** ^1^Department of Geriatrics, Zhongjiang County People’s Hospital, Deyang, China; ^2^Medical Records Management Department, Zhongjiang County People’s Hospital, Deyang, China; ^3^Hospital Infection Management Department, Zhongjiang County People’s Hospital, Deyang, China

**Keywords:** depression, hypertension, older adult patients, all-cause mortality, cardiovascular risk, meta-analysis, systematic review, mental health screening

## Abstract

**Background:**

Depression and hypertension frequently coexist in the older adult and may jointly contribute to increased mortality and cardiovascular risk. However, the extent to which depression independently affects these outcomes remains unclear. This systematic review and meta-analysis aimed to evaluate the association between depression and all-cause mortality in older adult patients with hypertension.

**Methods:**

We systematically searched PubMed, Embase, Cochrane Library, and Web of Science (2010–2024) for relevant cohort studies and randomized controlled trials. Pooled hazard ratios (HRs) were calculated using a random-effects model. Subgroup and sensitivity analyses were performed. The study protocol was registered in PROSPERO (CRD420251019904).

**Results:**

Thirteen studies including 483,560 participants showed that depression was associated with increased all-cause mortality (HR = 1.32, 95% CI: 1.23–1.41). The association was stronger among females (HR = 1.57) and in studies with short-term follow-up (<10 years, HR = 1.40). The findings were consistent across different depression assessment tools.

**Conclusion:**

Depression is independently associated with higher all-cause mortality in older adult hypertensive patients. Routine screening and management of depression—particularly among older women—may improve long-term outcomes. Further interventional studies are needed to evaluate the prognostic impact of depression treatment in this population.

**Systematic review registration:**

The systematic review was registered with PROSPERO (CRD420251019904).

## 1 Introduction

### 1.1 Research background

Hypertension and depression represent two critical global health concerns. Hypertension is a major modifiable risk factor for cardiovascular disease, the leading cause of death worldwide. Its prevalence and clinical management in older adult populations are particularly challenging due to physiological vulnerability, multimorbidity, and polypharmacy (1). Depression affects over 300 million individuals worldwide, substantially reducing quality of life and aggravating chronic illnesses, particularly in older adult. Increasing evidence suggests a bidirectional and complex interplay between hypertension and depression, highlighting the importance of considering their joint impact on patient outcomes (2, 3). For instance, depressive symptoms can contribute to the development and poor management of hypertension, and in turn, hypertension can exacerbate depressive states (1, 3). Specifically, studies such as the Hypertension in the Very Older adult Trial (HYVET) have shown that older adult individuals with hypertension and depressive symptoms have higher risks of cardiovascular morbidity and mortality, as well as cognitive decline (1).

Recent epidemiological studies have provided additional support for this association. Almeida et al. demonstrated that depression significantly elevates the risk of cardiovascular events and mortality in older men (2). Furthermore, the SPRINT trial indicated that depressive symptoms in hypertensive patients are strongly correlated with adverse clinical outcomes, including all-cause mortality (4). Other cohort studies have found consistent results among diverse populations, highlighting mood disorders as important determinants of cardiometabolic health trajectories and life expectancy in older adult populations (3, 5, 6). Given these findings, it is evident that the interaction between depression and hypertension profoundly influences health outcomes in older adult.

Although several prior meta-analyses have investigated the association between depression and mortality among hypertensive patients, important gaps remain. Many previous studies have limitations such as short follow-up durations, inconsistent adjustment for confounding factors, and a lack of comprehensive subgroup analyses (e.g., by gender or follow-up period). Additionally, few studies have focused specifically on older adult populations, who may have unique clinical characteristics and higher vulnerability. Therefore, our review aims to address these gaps by synthesizing the most recent evidence, incorporating detailed subgroup and sensitivity analyses, and providing updated insights into the impact of depression on mortality in older adult hypertensive patients.

### 1.2 Significance of the study

Despite accumulating evidence, the relationship between depression and hypertension in older adult populations remains inadequately understood, particularly concerning long-term prognosis and underlying mechanisms. Older adult frequently experience multimorbidity and require complex medication regimens, conditions that may influence both cardiovascular outcomes and overall quality of life (3, 5). Thus, elucidating how depression exacerbates hypertension-related complications through physiological, psychological, and behavioral pathways is critically important. Comprehensive management strategies addressing depression could significantly impact cardiovascular health, potentially reducing the risk of mortality and morbidity in this high-risk population.

### 1.3 Objectives of the study

This study aims to systematically evaluate the impact of depression on the long-term prognosis of older adult patients with hypertension, specifically focusing on all-cause mortality and cardiovascular risks. The primary objectives of this study are as follows:

(1) To comprehensively evaluate the association of depression with increased risks of all-cause mortality and cardiovascular events in older adult hypertensive patients, clarifying the clinical implications of their coexistence on health outcomes.
(2) To discuss possible biological, psychological, and behavioral mechanisms underlying the adverse effects of depression on cardiovascular health, emphasizing how effective depression management strategies (including pharmacological interventions and lifestyle modifications) may improve cardiovascular outcomes and survival in this vulnerable population.
(3) To propose potential clinical practices and policy recommendations based on the findings, aiming to enhance quality of care and health outcomes for older adult individuals suffering from both hypertension and depression.

## 2 Methods

### 2.1 Study design

This study was designed and reported following the PRISMA guidelines. By synthesizing existing research data, this review aims to offer a new perspective on how mental health status influences the health outcomes of older adult hypertensive patients.

The protocol for this study was prospectively registered in PROSPERO (CRD420251019904), an international database of prospectively registered systematic reviews.

To clarify the scope and eligibility of this systematic review, we formulated the review question using the PECOS framework: Population (P):** Older adult patients (aged ≥60 years) with hypertension; Exposure (E): Depression or depressive symptoms, as assessed by clinical diagnosis or validated scales; Comparator (C): Older adult hypertensive patients without depression; Outcomes (O): All-cause mortality, cardiovascular mortality, and major adverse cardiovascular events; Study Design (S): Cohort studies (prospective or retrospective), randomized controlled trials, and longitudinal studies.

### 2.2 Literature search

To comprehensively collect studies evaluating the impact of depression and hypertension on cardiovascular health in older adult populations, we systematically searched the following databases: PubMed, Embase, Cochrane Library, and Web of Science. The search period was restricted to 2010 to 2024 in order to capture the most up-to-date treatment strategies, diagnostic criteria, and research methodologies related to hypertension and depression. This timeframe also aligns with the publication of the majority of large-scale cohort studies and registry data in this field.

This approach aimed to enhance the clinical relevance and consistency of the included studies. Only English-language studies were included due to their typically rigorous peer-review standards and broader international recognition. Although these restrictions might introduce potential language and time-related biases, we believe this approach ensures higher consistency, relevance, and methodological quality of the studies included. The search terms included: (“Hypertension” or “High blood pressure” or “Essential hypertension”) and (“Depression” or “Depressive Disorder” or “Depressive symptoms” or “Major depression” or “Mental health”) and (“Prognosis” or “Mortality” or “Survival Analysis” or “Cardiovascular Events” or “Long-term outcome” or “All-cause mortality” or “Major Adverse Cardiovascular Events” or “MACE”) and (“Cohort Studies” or “Longitudinal Studies” or “Follow-Up Studies” or “Prospective Studies” or “Retrospective Studies” or “Randomized Controlled Trial” or “RCT”) and (“Aged” or “Aged, 80 and over” or “Older adult” or “Older adult” or “Geriatric” or “Aging population” or “≥60 years”).

### 2.3 Study selection

Five reviewers independently screened titles and abstracts, retrieved full texts, and selected eligible studies according to the predefined PECOS framework. The inclusion criteria were as follows:

(1) Population: Older adult patients diagnosed with hypertension who had undergone depression assessment;
(2) Exposure: Depression or depressive symptoms (measured by validated scales or clinical diagnosis);
(3) Comparator: Hypertensive patients without depression;
(4) Outcomes: All-cause mortality, cardiovascular mortality, stroke-related mortality, and other long-term prognostic indicators;
(5) Study design: Prospective cohort studies, retrospective cohort studies, randomized controlled trials (RCTs), and high-quality meta-analyses.

Exclusion criteria: Studies were excluded if they focused on non-older adult populations, did not assess depression in hypertensive patients, examined only short-term blood pressure fluctuations, or were case reports, conference abstracts, review articles, commentaries, or had incomplete data or insufficient information to calculate effect estimates.

### 2.4 Data extraction and quality assessment

To ensure data accuracy and study objectivity, four reviewers independently performed data extraction and quality assessment, with all procedures supervised by the first author. Any disagreements were resolved through team discussions to reach a consensus. The following data were extracted from eligible studies: first author, year of publication, study region, study design type, follow-up duration, sample size, mean age of participants, gender distribution, depression assessment tools, primary study outcomes, hazard ratio (HR) with 95% confidence interval (CI) and *p*-value, as well as covariates considered for adjustment.

**Figure 1:**
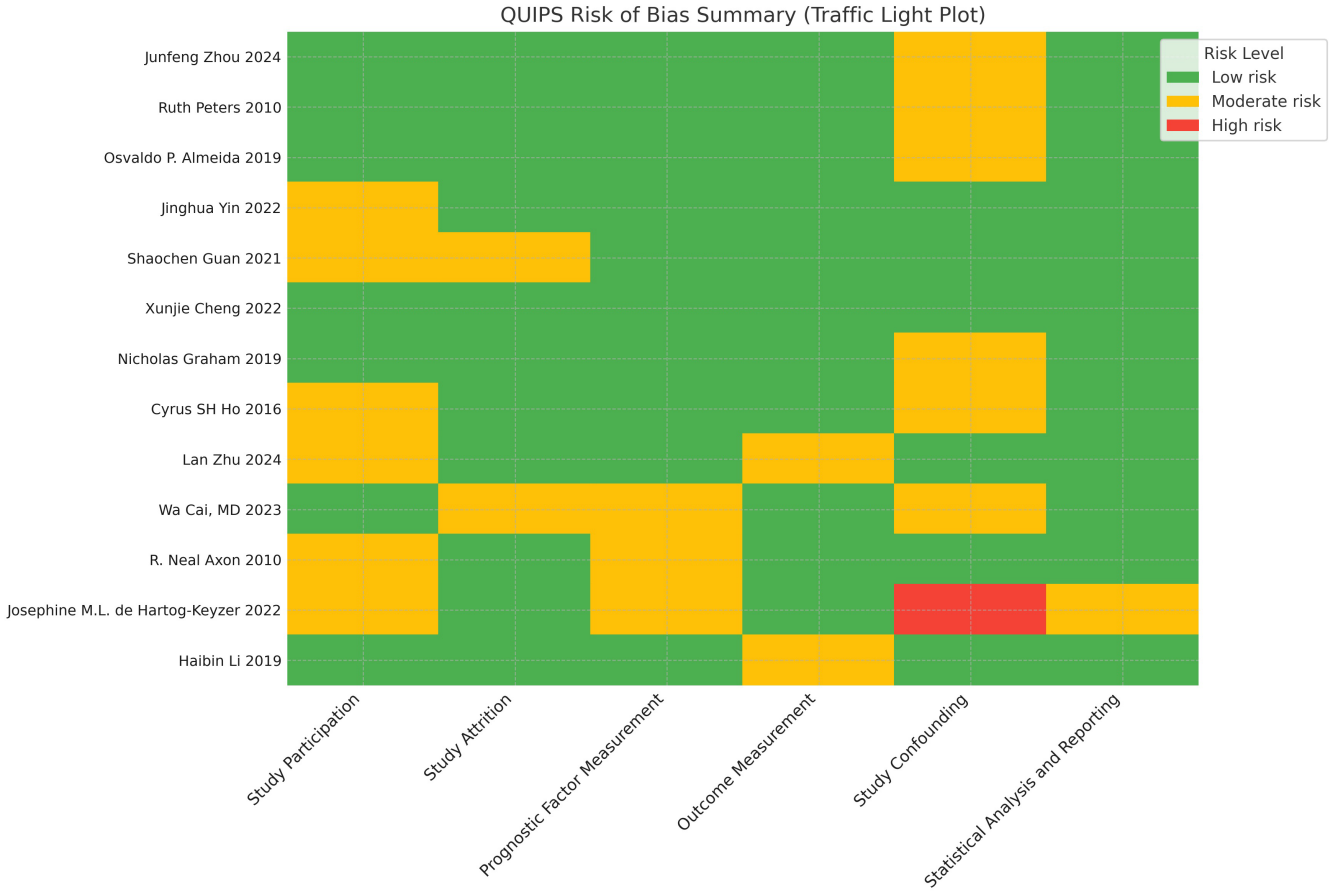
Traffic light plot of risk of bias assessment using the QUIPS tool across all included studies. Green, low risk of bias; yellow, moderate risk of bias; red, high risk of bias.

We used the Cochrane Risk of Bias Tool to assess the quality of randomized controlled trials (RCTs), categorizing studies into low risk of bias, some concerns, and high risk of bias. For cohort studies, we adopted the Quality in Prognosis Studies (QUIPS) tool, which evaluates six domains: study participation, study attrition, prognostic factor measurement, outcome measurement, study confounding, and statistical analysis and reporting. Each domain was judged as having a low, moderate, or high risk of bias. A traffic light plot was generated to visualize the risk-of-bias judgments across all included studies (Figure 1).

### 2.5 Statistical analysis

All statistical analyses were performed using Stata 18.0 software.

First, study results were pooled to assess the association between depression and mortality in older adult hypertensive patients. To evaluate heterogeneity across studies, Cochran’s Q test and I^2^ statistics were applied. Given the significant clinical heterogeneity of depression, all analyses were conducted using a random-effects model.

Second, to explore potential sources of heterogeneity, subgroup analyses were performed based on patient gender, sample size, follow-up duration, and depression assessment tools. Sensitivity analysis was conducted by sequentially excluding each study to evaluate the robustness of the overall risk estimate. Additionally, potential publication bias was assessed using funnel plots, Begg’s rank correlation test, and Egger’s regression test. The symmetry of the funnel plot was examined visually, while Begg’s and Egger’s statistical tests were used to quantify the potential impact of small-study effects. A statistical significance level of *p* < 0.05 was set for all tests.

Finally, the GRADE (Grading of Recommendations, Assessment, Development, and Evaluations) approach was employed to comprehensively assess the quality of evidence, ensuring the credibility and applicability of the study findings.

## 3 Results

### 3.1 Literature search results

**Figure 2:**
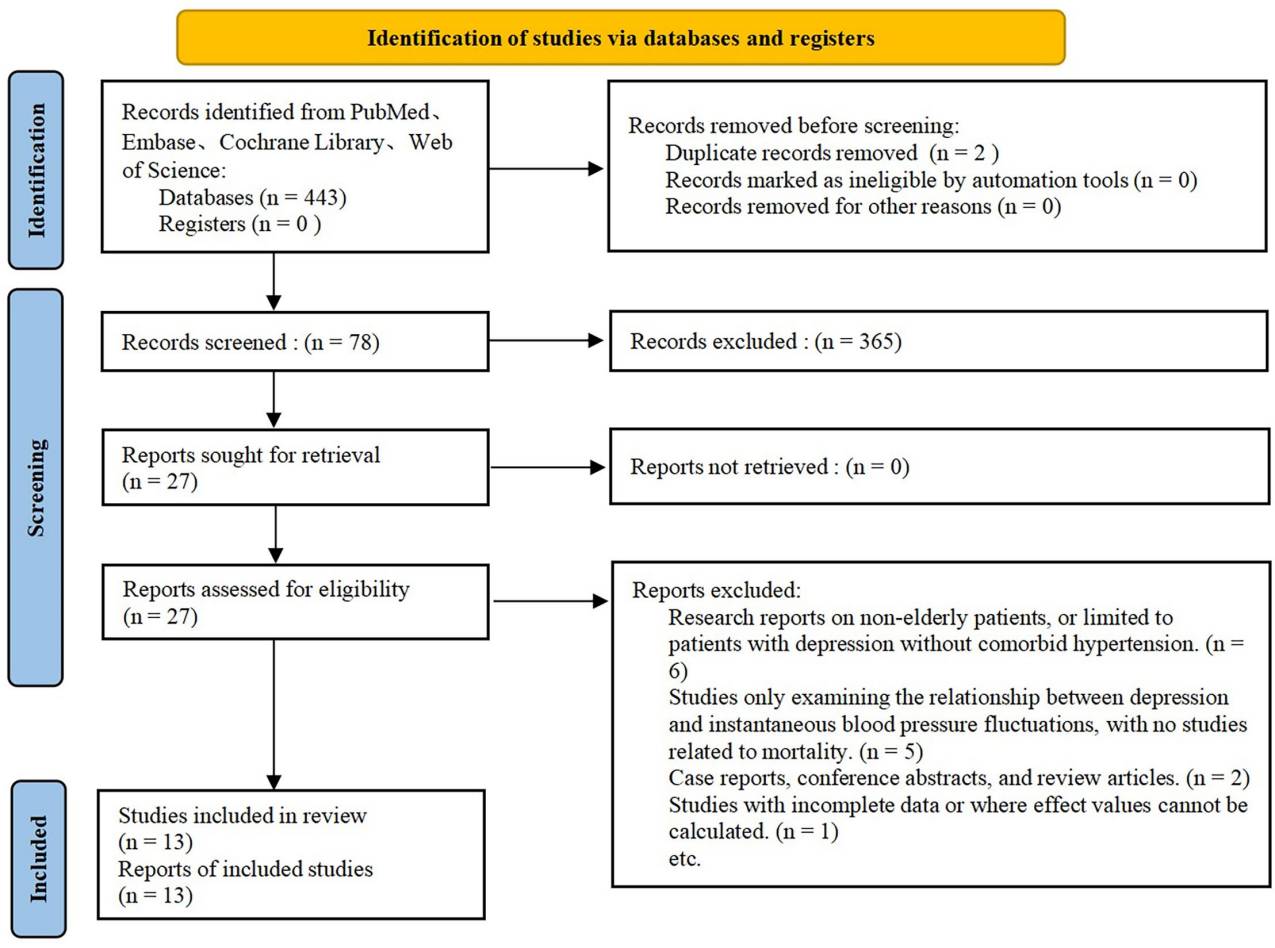
PRISMA flow diagram of study selection.

A total of 443 potentially relevant studies were identified through the initial database search. After removing duplicates and screening titles and abstracts, 78 studies were selected for full-text review. Following a detailed evaluation, 65 studies were excluded based on the predefined exclusion criteria. Finally, 2 randomized controlled trials and 11 cohort studies (1–13) were included in the final meta-analysis. The study selection process is detailed in Figure 2.

### 3.2 Characteristics of included studies

The characteristics of the included studies are summarized in Table 1. These studies were published between 2010 and 2024 and were conducted across multiple countries and regions. The total number of participants covered in the included studies was 483,560, with individual study sample sizes ranging from 555 to 261,297. The follow-up period varied between 2.1 years and 13.3 years. The included studies reported outcomes such as all-cause mortality, cardiovascular mortality and fatal stroke.

**Table 1:** Hypertension depression mortality data.

Author/year	Region	Design	Follow-up (years)	Sample size (% women)	Average age (years)	Depression assessment tools	Outcome	HR (95% CI)	*p*-value	Adjustment factor
Peters et al. (2010) (1)	China, United Kingdom	RCT	2.1	2,656 (60.6)	83.5	GDS-15	All-cause mortality.	1.18 (0.91–1.67)	<0.001	Age, gender, treatment group, country/region, educational level, living alone, number of comorbidities, CVD.
Almeida et al. (2019) (2)	Australia	Cohort Study	12	5,522 (0.0)	77.3	GDS-15	All-cause mortality.	1.41 (1.20–1.67)	*p* < 0.001	Age, antiplatelet drug use, diabetes, hypertension(HTN), coronary heart disease(CHD), stroke, chronic respiratory diseases, dementia, and digestive and kidney diseases.
Yin et al. (2022) (5)	United Kingdom	Cohort Study	12.3	95,351 (48.7)	≥60	ICD-10	All-cause mortality.	1.17 (0.94–1.34)	*p* < 0.001	Gender, age, race, Townsend Deprivation Index, body mass index(BMI), smoking status, alcohol consumption, diet, physical activity, and history of cancer.
Guan et al. (2021) (3)	China	Cohort Study	4.97	863 (100.0)	70.1	CES-D	All-cause mortality.	1.68 (1.25–2.16)	*p* = 0.013	Age, marital status, smoking, alcohol consumption, educational level, physical activity, activities of daily living (ADL), and self-rated income.
Cheng et al. (2022) (6)	United Kingdom	Cohort Study	10.9	261,297 (56.6)	≥60	ICD-10	All-cause mortality.	1.12 (0.85–1.33)	*p* = 0.002	Age, gender, race, Townsend Deprivation Index, family history of cardiovascular disease, body mass index (BMI), smoking, alcohol consumption, diet, physical activity, and sedentary time.
Graham et al. (2019) (7)	United Kingdom	Cohort Study	5.25	12,929 (59.4)	60	ICD-10	All-cause mortality. Cardiovascular mortality.	1.66 (1.25–1.81)	*p* < 0.001	Age, gender, Townsend Deprivation Index, years of education, race, history of diabetes, hypercholesterolemia, body mass index (BMI), smoking, alcohol consumption, physical activity, and use of psychotropic drugs.
Zhu et al. (2024) (8)	China	Cohort Study	10	13,855 (60.4)	≥60	CES-D	All-cause mortality.	1.32 (1.08–1.49)	*p* < 0.001	Age, gender, household registration type, marital status, educational level, income, pension, occupation, smoking, alcohol consumption, regular eating habits, nap habits, and history of chronic diseases.
Axon et al. (2010) (9)	United States	Cohort Study	8	10,025 (62.8)	≥60	CES-D	All-cause mortality.	1.39 (1.14–1.69)	*p* < 0.001	Age, gender, race, body mass index (BMI), marital status, educational level, income, physical activity, smoking, aspirin use, diabetes, cancer, stroke, and coronary heart disease (CHD).
de Hartog-Keyzer et al. (2022) (10)	Netherlands	Cohort Study	8	555 (56.0)	70	PHQ-9	All-cause mortality.	1.18 (0.88–1.48)	*p* < 0.0001	Age, gender, smoking, cholesterol levels, systolic blood pressure, body mass index (BMI), history of coronary heart disease (CHD), and use of antihypertensive drugs.
Li et al. (2019) (11)	China	Cohort Study	4	1,2,417 (50.8)	≥60	CES-D	All-cause mortality.	1.39 (1.22–1.58)	P < 0.001	Age, gender, place of residence, marital status, educational level, smoking, alcohol consumption, diabetes, hypertension, dyslipidemia, history of chronic kidney disease, and body mass index (BMI).
Zhou et al. (2024) (4)	China	Cohort Study	13.3	9,259 (35.65)	67.9	PHQ-9	All-cause mortality.	1.29 (0.89–1.48)	*p* = 0.0038	Age, gender, body mass index (BMI), hyperlipidemia, antihypertensive medication use, and smoking history.
Ho et al. (2016) (12)	Singapore	Cohort Study	10	1,070 (55.45)	≥60	GMS	All-cause mortality.	1.39 (1.22–1.58)	*p* = 0.15	Age, gender, race, marital status, health behaviors (smoking, alcohol consumption, physical activity), and physical health comorbidities.
Cai et al. (2023) (13)	Multiple countries	SR/MA	12	57,761(No data)	≥60	CES-D	All-cause mortality. Fatal stroke.	1.03 (0.71–1.35)	*p* < 0.001	Age, gender, education, smoking status, and comorbidities.

The tools used to assess depression included the 15-item Geriatric Depression Scale (GDS-15), the Center for Epidemiologic Studies Depression Scale (CES-D), the Patient Health Questionnaire-9 (PHQ-9), the Geriatric Mental State Examination (GMS), and the International Classification of Diseases, 10th Revision (ICD-10) diagnosis.

Risk of bias assessments were performed for all included studies. The two randomized controlled trials were evaluated using the Cochrane Risk of Bias Tool and were classified as having some concerns. For the 13 included cohort studies, the Quality in Prognosis Studies (QUIPS) tool was used to assess domain-specific risks of bias. While most studies were rated as low risk in prognostic factor and outcome measurements, moderate risk was frequently identified in confounding and participation domains. Details are provided in Supplementary Text 1 and Figure 1.

### 3.3 Effect of depression on mortality in older adult hypertensive patients

**Figure 3:**
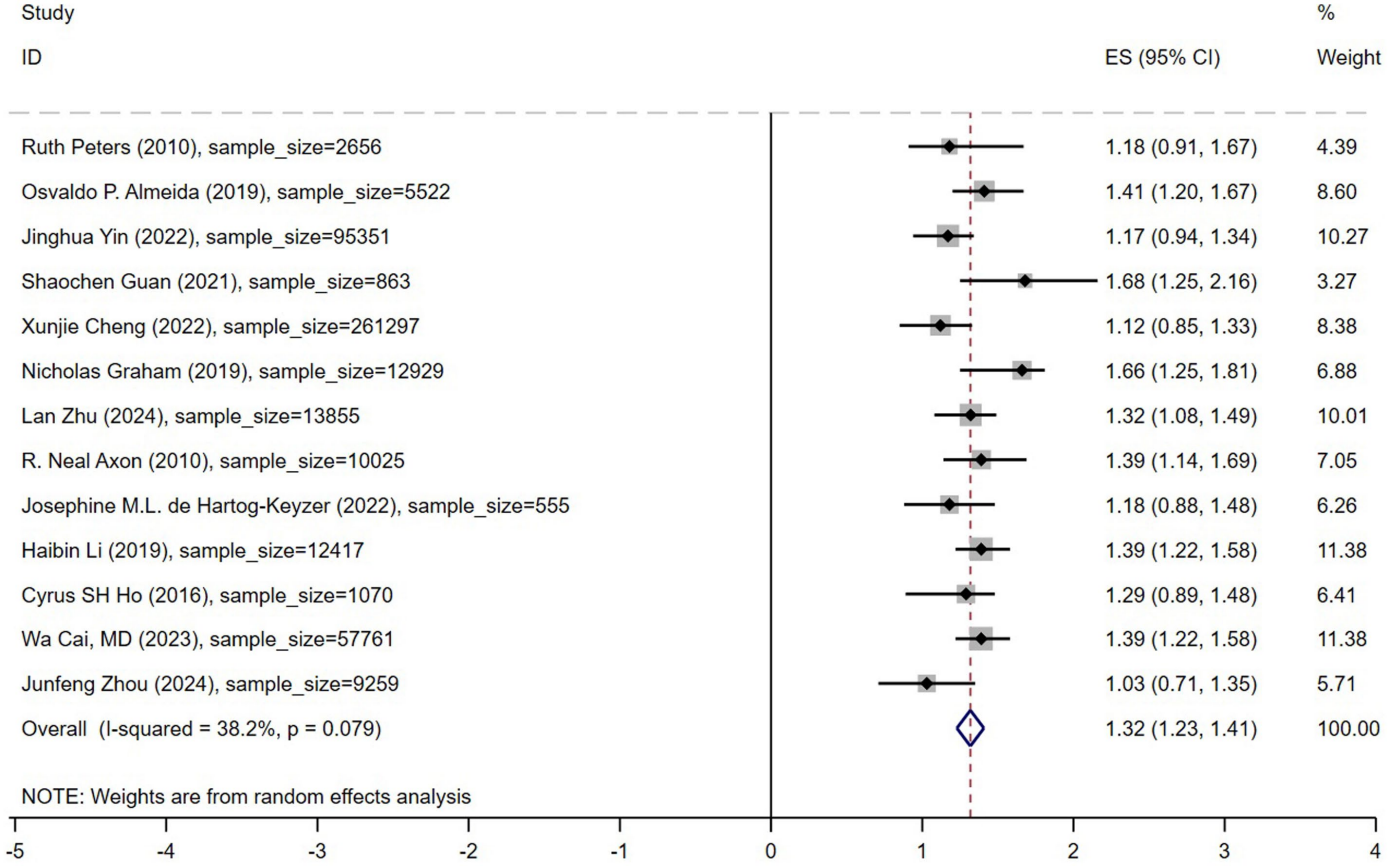
Forest plot depression hypertension mortality.

A random-effects model was used for meta-analysis, and the results are presented in Figure 3. The analysis showed that depression was significantly associated with an increased the risk of mortality in older adult hypertensive patients, with a pooled hazard ratio (HR) of 1.32 (95% confidence interval: 1.23–1.41). As illustrated in the figure, 11 out of the 13 included studies reported an HR greater than 1, indicating an increased mortality risk associated with depression. Among these, four studies had 95% confidence intervals that did not include 1, suggesting statistically significant effects of depression on mortality. The heterogeneity among studies was moderate (*I*^2^ = 38.2%, *p* = 0.079), indicating some degree of variability between studies while maintaining an overall consistent trend. The sample sizes across studies ranged from 555 to 261,297, ensuring the representativeness of this meta-analysis. These findings suggest that depression is associated with an increased mortality risk among older adult hypertensive patients. Therefore, depression screening and psychological interventions should be incorporated into hypertension management strategies to mitigate adverse prognostic outcomes.

**Figure 4:**
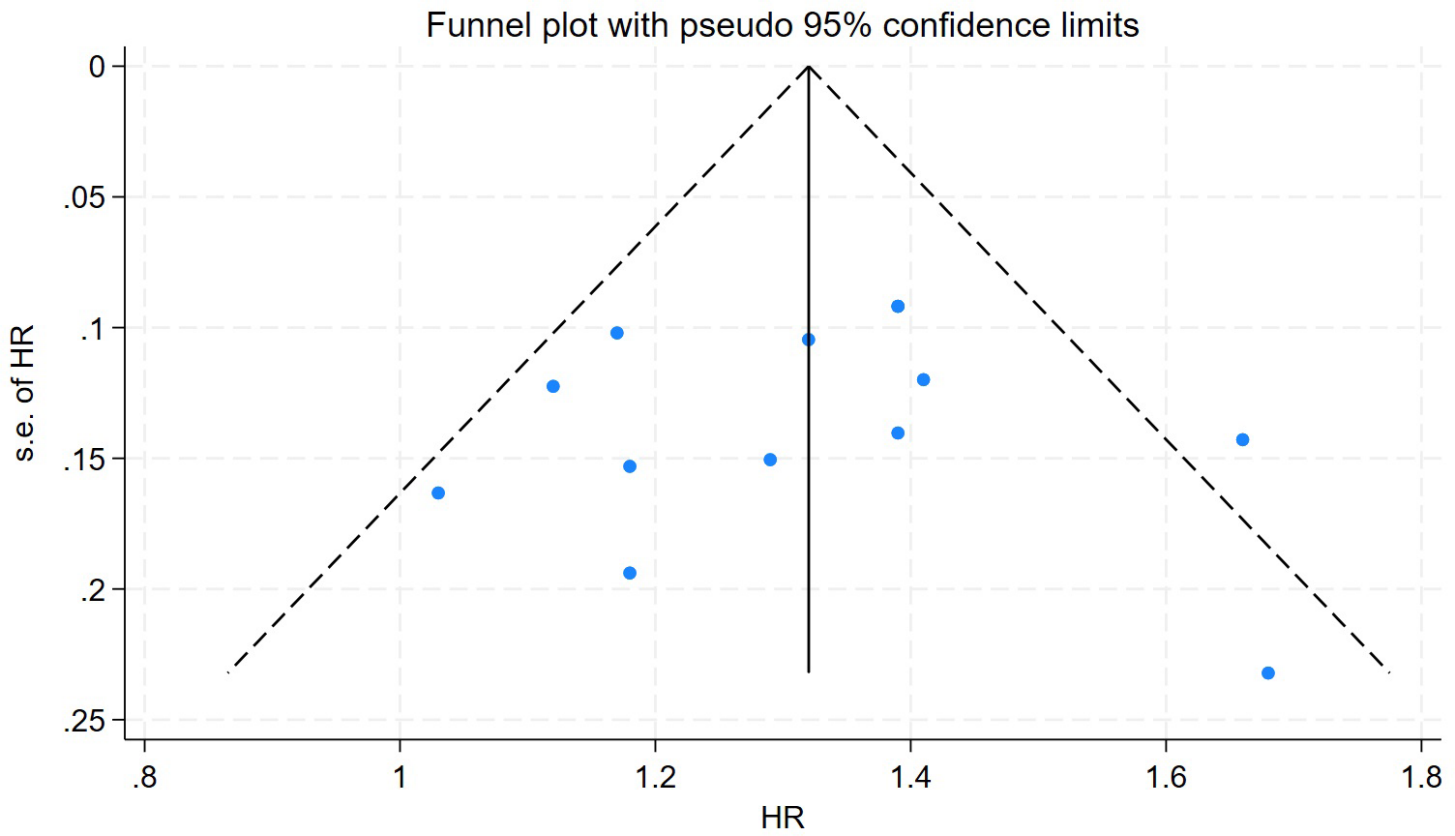
Funnel plot for publication bias assessment.

To assess potential publication bias, a funnel plot was generated (Figure 4). The funnel plot exhibited slight asymmetry, particularly with fewer studies observed in the lower left region, which may indicate the presence of potential publication bias. However, statistical tests for publication bias, including Begg’s test (*p* = 0.428) and Egger’s test (*p* = 0.985), did not reveal statistically significant bias. This suggests that, despite some asymmetry in the funnel plot, publication bias is unlikely to have a substantial impact on the overall findings of this study.

### 3.4 Subgroup analysis and sensitivity analysis

To explore the sources of heterogeneity, subgroup analyses were conducted based on gender, sample size, follow-up duration, and depression assessment methods (Table 2).

**Table 2:** Subgroup analysis data.

Subgroup	Number of studies	Pooled hazard ratio	95% confidence intervals	Heterogeneity between studies
Gender				
Men	11	1.15	(0.98–1.33)	*I*^2^ = 87.9% *p* = 0.000
Women	11	1.57	(1.28–1.86)	*I*^2^ = 91.8% p = 0.000
Sample_size				
>10,000	7	1.34	(1.22–1.45)	*I*^2^ = 49.0% *p* = 0.067
<10,000	6	1.32	(1.12–1.44)	*I*^2^ = 30.4% *p* = 0.207
Follow_up				
≥10 years	7	1.27	(1.17–1.37)	*I*^2^ = 24.4% *p* = 0.243
<10 years	6	1.4	(1.25–1.56)	*I*^2^ = 38.5% *p* = 0.149
Depression assessment tool				
GDS-15	2	1.35	(1.14–1.55)	*I*^2^ = 1.8% *p* = 0.313
ICD-10 Diagnosis	3	1.31	(1.00–1.61)	*I*^2^ = 79.8% *p* = 0.007
CES-D	5	1.34	(1.20–1.49)	*I*^2^ = 35.5% *p* = 0.184
PHQ-9	2	1.24	(1.03–1.45)	*I*^2^ = 0.0% *p* = 0.608

#### 3.4.1 Gender differences

**Figure 5:**
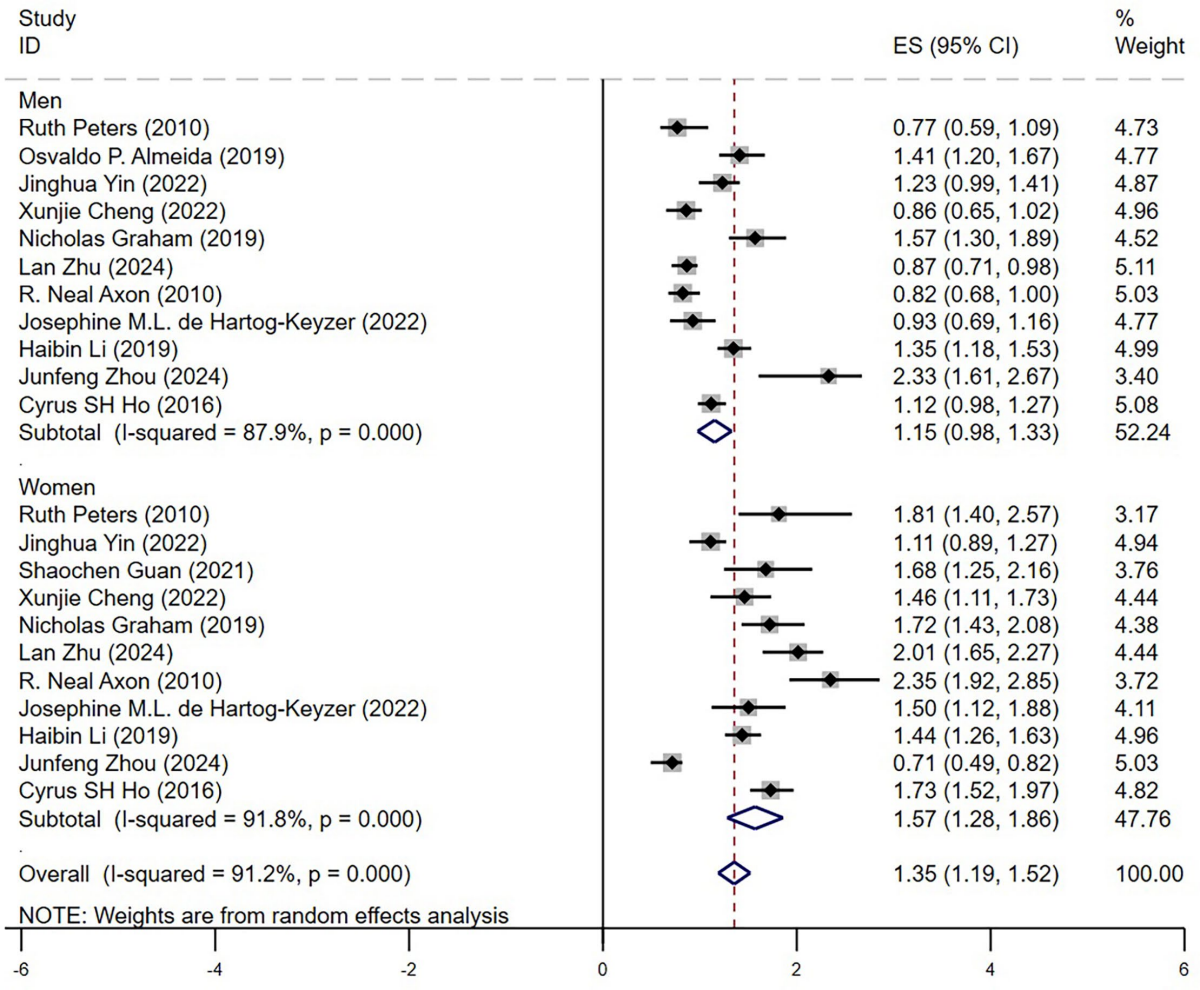
Forest plot gender.

In the gender subgroup analysis (Figure 5), depression was associated with a significantly higher mortality risk in female older adult hypertensive patients (HR = 1.57, 95% CI: 1.28–1.86, *p* < 0.05), whereas the risk increase in male patients was smaller and did not reach statistical significance (HR = 1.15, 95% CI: 0.98–1.33, *p* > 0.05). This finding indicates that older adult female patients may be more vulnerable to the adverse impact of depression, potentially due to physiological and psychological factors, including inflammatory responses, hormonal fluctuations, and differences in psychosocial stressors. Substantial heterogeneity was observed across studies (*I*^2^ = 87.9% in males, *I*^2^ = 91.8% in females), indicating considerable variability in the results.

#### 3.4.2 Sample size influence

**Figure 6:**
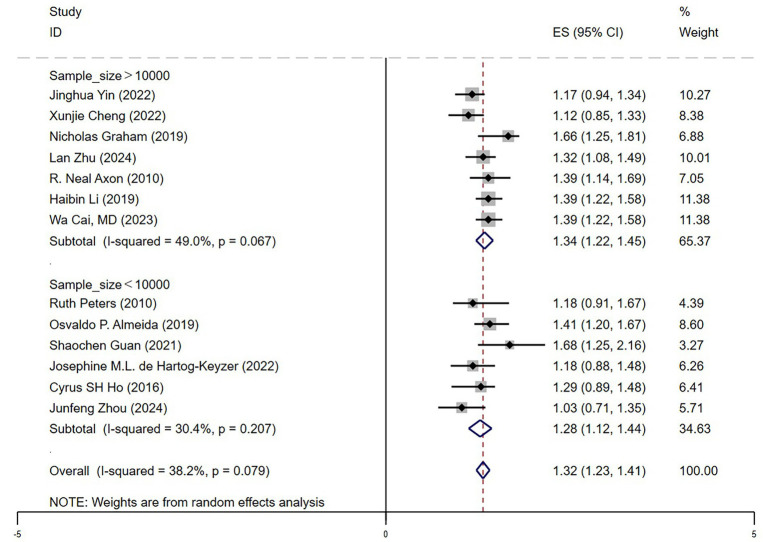
Forest plot sample size.

In the sample size subgroup analysis (Figure 6), depression was found to significantly increase mortality risk in older adult hypertensive patients, irrespective of study size. The pooled hazard ratio (HR) was 1.34 (95% CI: 1.22–1.45) in large-sample studies (*n* > 10,000) and 1.28 (95% CI: 1.12–1.44) in small-sample studies (*n* < 10,000). Heterogeneity was lower in large-sample studies (*I*^2^ = 49.0%), indicating greater consistency across studies and higher statistical power. Although small-sample studies exhibited even lower heterogeneity (*I*^2^ = 30.4%), the effect size (HR = 1.28) was slightly reduced, potentially reflecting statistical fluctuations in individual studies. Overall, these findings reinforce the conclusion that depression significantly increases mortality risk in older adult hypertensive patients, with large-sample studies providing more representative and robust evidence to inform clinical management.

#### 3.4.3 Follow-up duration impact

**Figure 7:**
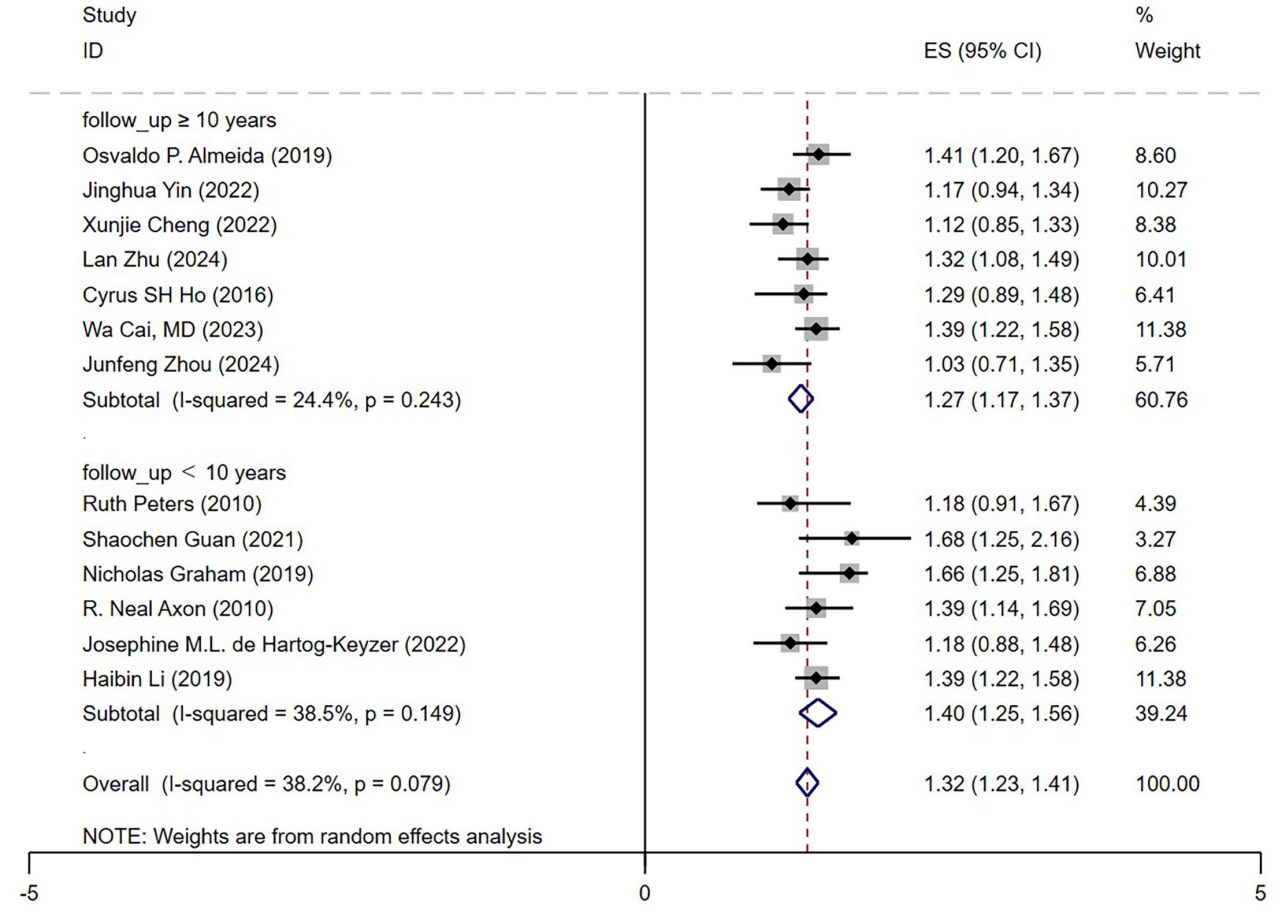
Forest plot follow up.

In the follow-up duration subgroup analysis (Figure 7), depression was found to significantly increase mortality risk in older adult hypertensive patients, irrespective of follow-up length. The pooled hazard ratio (HR) was 1.40 (95% CI: 1.25–1.56) for short-term follow-up (<10 years) and 1.27 (95% CI: 1.17–1.37) for long-term follow-up (≥10 years). The higher HR observed in short-term studies suggests that the impact of depression on mortality risk may be more pronounced in the early stages, potentially due to acute cardiovascular events or other comorbidities. Notably, long-term studies exhibited lower heterogeneity (I^2^ = 24.4%), indicating greater consistency across studies. Overall, these findings demonstrate that the detrimental effect of depression on mortality risk persists beyond the short term, remaining significant even in follow-ups exceeding 10 years. This underscores the critical importance of sustained psychological health management in older adult hypertensive patients.

#### 3.4.4 Depression assessment methods

**Figure 8:**
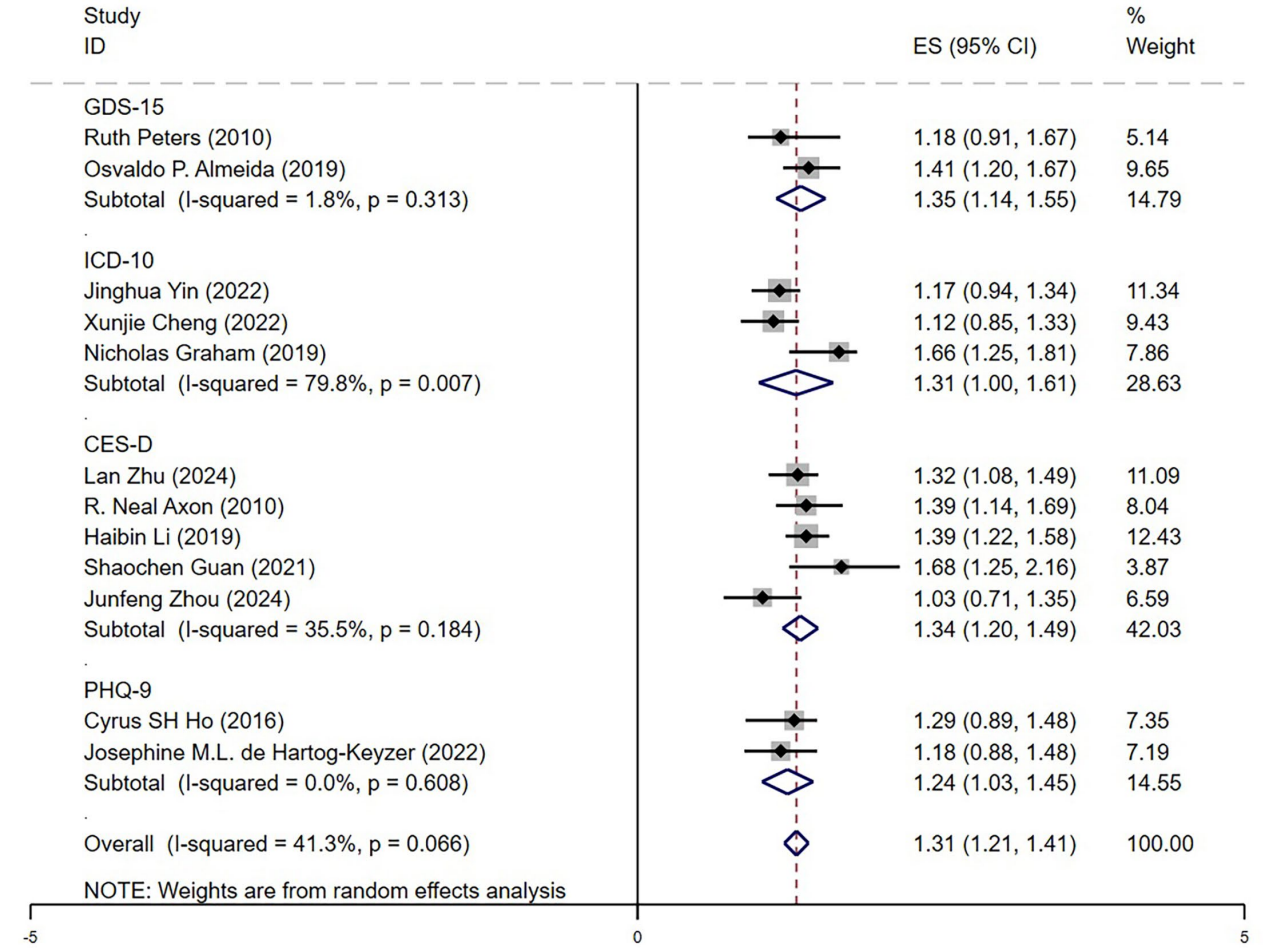
Forest plot depression scale.

In the depression assessment tool subgroup analysis (Figure 8), we found that depression significantly increased mortality risk in older adult hypertensive patients, regardless of the assessment tool used. The pooled hazard ratios (HRs) ranged from 1.24 (PHQ-9) to 1.35 (GDS-15). The GDS-15 and CES-D scales showed higher HR values (HR = 1.35 and 1.34, respectively), suggesting that these tools were more sensitive in capturing the impact of depression. Conversely, ICD-10 diagnosis had a slightly lower HR (HR = 1.31) but the highest heterogeneity among studies (*I*^2^ = 79.8%), indicating potential variations in diagnostic criteria across studies. The PHQ-9 was associated with the lowest heterogeneity (*I*^2^ = 0%), suggesting high consistency in its measurement results. Overall, the HR values across different assessment tools were similar, confirming that the effect of depression on mortality risk in older adult hypertensive patients was robust. These findings further emphasized the importance of clinical psychological health management in this population.

#### 3.4.5 Sensitivity analysis

To verify the robustness of our study conclusions, we conducted the following sensitivity analyses:

(1) Recalculation of overall HR after excluding the high-heterogeneity subgroup (ICD-10): The results showed HR = 1.32 (95% CI: 1.22–1.42, *I*^2^ = 29.5%), indicating a decrease in heterogeneity. This suggests that standardization issues within the ICD-10 subgroup may have contributed to the overall heterogeneity.
(2) Including only large-sample studies (*n* > 10,000): The results showed HR = 1.33 (95% CI: 1.20–1.47, *I*^2^ = 38.2%), indicating that the association remained significant. This suggests that excluding small-sample studies did not affect the overall trend, confirming the robustness of the findings.
(3) Fixed-effects vs. random-effects model: The random-effects model yielded HR = 1.31 (95% CI: 1.21–1.41), while the fixed-effects model produced HR = 1.29 (95% CI: 1.22–1.35). The slightly lower HR in the fixed-effects model suggests that the random-effects model may slightly overestimate the effect size, but the association remains significant, confirming the robustness of the findings.
(4) Excluding studies with follow-up <5 years: The results showed HR = 1.30 (95% CI: 1.18–1.41), indicating that excluding short-term follow-up studies did not affect the study conclusions. This suggests that the impact of depression on mortality remains stable over the long term.

Comprehensive analysis: Depression significantly increases mortality risk in older adult hypertensive patients, a trend consistently observed across all subgroups. Female patients exhibited a higher risk, emphasizing the need for closer psychological health monitoring in this population. Large-sample and long-term follow-up studies provided more robust evidence, further enhancing the credibility of the study conclusions. Variations in depression assessment tools may influence study outcomes, with higher heterogeneity observed in studies using ICD-10 diagnostic criteria, whereas assessments based on GDS-15 and PHQ-9 demonstrated greater consistency. Sensitivity analyses confirmed the robustness of the findings—regardless of excluding high-heterogeneity studies, adjusting follow-up durations, or modifying statistical models, the association between depression and increased mortality risk remained stable.

### 3.5 GRADE evidence quality

The overall GRADE assessment indicated that the quality of evidence was moderate (detailed information available in Supplementary Text 2).

## 4 Discussion

### 4.1 Mechanisms underlying the association between depression and long-term prognosis in hypertensive patients

Depression is associated with increased all-cause mortality in older adult hypertensive patients. This association is likely driven by physiological and behavioral mechanisms, such as autonomic dysfunction, chronic inflammation, and poor adherence to treatment.

(1) Sympathetic Nervous System (SNS) Overactivation: Depression activates the sympathetic nervous system (SNS), increasing cardiovascular stress and worsening hypertension (4). Additionally, heightened sympathetic tone can increase platelet activation and elevate the risk of arrhythmias, further contributing to higher cardiovascular mortality (3).
(2) Hyperactivation of the Hypothalamic–Pituitary–Adrenal (HPA) Axis: HPA axis overactivation in depression elevates cortisol, promoting insulin resistance, endothelial dysfunction, and hypertension (4).
(3) Chronic Inflammation and Endothelial Dysfunction: Depressed patients often exhibit elevated levels of systemic inflammation, characterized by increased C-reactive protein (CRP), interleukin-6 (IL-6), and tumor necrosis factor-alpha (TNF-*α*). These inflammatory markers contribute to endothelial damage, lipid deposition, and atherosclerosis, thereby increasing the risk of cardiovascular events in hypertensive patients^3^. Furthermore, chronic inflammation can lead to increased vascular stiffness, further exacerbating the progression of hypertension (4).
(4) Poor Health Behaviors and Medication Non-Adherence: Depressed patients often exhibit poor health behaviors, such as low adherence to antihypertensive medications, which has been shown to be significantly lower in depressed individuals compared to non-depressed patients, leading to poor blood pressure control^4^. Depression is also associated with unhealthy dietary habits, including high sugar and high-fat intake, which may increase the risk of metabolic syndrome (3). Additionally, depressed patients tend to engage in less physical activity, resulting in weight gain, worsening insulin resistance, and further deterioration of cardiovascular health.

In summary, depression exacerbates the long-term prognosis of hypertensive patients through multiple mechanisms, including sympathetic nervous system activation, HPA axis dysregulation, chronic inflammatory states, and unhealthy lifestyle behaviors.

In addition to increased all-cause mortality, several studies also reported a higher risk of fatal stroke in depressed older adult hypertensive patients. This elevated risk may be explained by similar pathophysiological pathways, including systemic inflammation, vascular endothelial dysfunction, and platelet hyperreactivity. These processes can predispose individuals to acute cerebrovascular events, further underscoring the importance of mental health management in this population.

This finding is consistent with a recent meta-analysis by Ashraf et al. (14), which demonstrated that depression significantly increases the risk of stroke, including ischemic, hemorrhagic, and fatal subtypes (HR = 1.41, 95% CI: 1.32–1.50), with variations by follow-up duration. Their findings underscore the broader vascular impact of depression and reinforce the importance of integrated mental health strategies in cardiovascular disease prevention. Our study extends this perspective by focusing specifically on all-cause mortality in older adult hypertensive patients, further highlighting the multifactorial burden of depression in this high-risk population.

### 4.2 The impact of gender on the association between depression and mortality risk

The association between depression and mortality appeared stronger in women than in men, which aligns with findings from previous studies. This may be due to hormonal changes post-menopause, which influence cardiovascular regulation, and increased psychosocial stress from loneliness and caregiving responsibilities (3, 4). These findings highlight the need for gender-specific interventions in hypertension and mental health care. This stronger association may also reflect cumulative effects of inflammation, autonomic dysregulation, and mood instability, which disproportionately affect older women.

### 4.3 Validation of the impact of depression based on follow-up duration and assessment methods

The association between depression and mortality risk persisted across both short-term and long-term follow-up periods. In our study, short follow-up (<10 years) showed a higher risk (HR = 1.40, 95% CI: 1.25–1.56), likely due to acute cardiovascular events and comorbidities, while long-term follow-up (≥10 years) confirmed a persistent, though slightly attenuated, effect (HR = 1.27, 95% CI: 1.17–1.37). These findings underscore the necessity of sustained psychological health management for hypertensive patients.

Despite variations in depression assessment tools (GDS-15, CES-D, ICD-10, PHQ-9), the overall trend remained consistent, confirming the robustness of findings. However, studies using ICD-10 showed the highest heterogeneity (*I*^2^ = 79.8%), highlighting the need for standardized diagnostic criteria in future research.

The observed heterogeneity across studies may, in part, be attributed to differences in depression assessment tools. Instruments such as PHQ-9 and CES-D are self-reported symptom scales with lower diagnostic thresholds and high sensitivity, which may identify milder or subclinical cases. In contrast, ICD-10 relies on structured clinical diagnosis and may capture more severe depression. These differences in diagnostic rigor and symptom threshold likely affect which participants are classified as depressed, thus contributing to variability in effect estimates. The high heterogeneity observed in the ICD-10 subgroup may also reflect inconsistencies in the application of diagnostic criteria across different healthcare settings.

### 4.4 Clinical implications

The findings of this study have significant clinical implications for hypertension management and cardiovascular disease prevention. While hypertension management traditionally focuses on blood pressure control, our results emphasize the need to integrate mental health assessment. Based on these findings, three key strategies warrant further attention:

(1) Routine Psychological Screening for Hypertensive Patients: Regular mental health screening may be beneficial for hypertensive patients, particularly in high-risk groups (e.g., women, those with poor medication adherence, and multimorbidity). Current guidelines rarely include psychological health assessments, but integrating depression screening tools such as PHQ-9, GDS-15, and CES-D into follow-ups may improve long-term outcomes (4).
(2) Psychological Interventions for High-Risk Depressed Patients: Cognitive behavioral therapy (CBT) has demonstrated effectiveness in alleviating depressive symptoms, reducing sympathetic nervous system excitability, and improving cardiovascular health (4). Additionally, medication non-adherence is prevalent among depressed patients, and psychological interventions can enhance self-management, improving blood pressure control and reducing cardiovascular risks (3). Integrating lifestyle modifications (e.g., exercise and diet) with psychological interventions may further benefit hypertensive patients. Future research should refine personalized intervention strategies.
(3) Further Research on the Impact of Antidepressant Treatment on Hypertension Prognosis: The cardiovascular effects of antidepressants vary. SSRIs may be relatively safe and improve vascular function, while TCAs and NDRIs could elevate sympathetic activity and blood pressure, increasing cardiovascular risk (2–4). Further research, particularly RCTs, is needed to determine the optimal antidepressant strategy for hypertensive patients with comorbid depression.

### 4.5 Public health and healthcare implications

Our findings suggest that integrating depression screening and treatment into hypertension management for older adult patients could help reduce both mortality and healthcare costs. Depression in this population increases healthcare utilization and resource burden. Early intervention may improve patient outcomes and lessen the financial strain on health systems. These results support multidisciplinary care models addressing both physical and mental health in older adult with hypertension.

### 4.6 Limitations of the study

While this meta-analysis confirmed a significant association between depression and increased mortality risk in older adult hypertensive patients, several limitations should be considered when interpreting these findings.

First, the included studies varied in study design, geographic regions, sample characteristics, and adjustment variables, contributing to heterogeneity despite the use of a random-effects model. Residual confounding cannot be ruled out, as unmeasured or inadequately adjusted variables—such as differences in depression diagnostic criteria, socioeconomic status, comorbidities, and antidepressant use—may have influenced the results. As the included studies are observational in nature, causality cannot be inferred from the reported associations. Future research should refine patient stratification and focus on more homogeneous populations to improve generalizability.

Second, differences in depression assessment tools may have introduced bias. While ICD-10 likely identifies more severe cases, CES-D and PHQ-9 capture a broader spectrum of depressive symptoms, leading to variability in effect estimates. The highest heterogeneity was observed in ICD-10 studies (I^2^ = 79.8%), highlighting the need for standardized diagnostic tools in future research.

Third, follow-up durations ranged from 2.1 to 13.3 years, with some studies having shorter observation periods, potentially limiting the ability to assess long-term effects. Future studies should prioritize long-term prospective cohorts to clarify depression’s sustained impact on hypertensive patients and mitigate potential biases from short-term stress responses.

Finally, while Begg’s (*p* = 0.428) and Egger’s (*p* = 0.985) tests did not indicate significant publication bias, the potential for selective reporting and underrepresentation of null results remains, as suggested by the slight funnel plot asymmetry. Studies with statistically significant findings may have been more likely to be published, while non-significant results could remain unpublished, warranting cautious interpretation of the findings. For further supporting literature, methodological details, and related evidence, see also references (15–40).

## 5 Conclusion

In summary, this systematic review and meta-analysis demonstrates that depression significantly increases mortality risk among older adult patients with hypertension. Our findings underscore the importance of integrating mental health screening and intervention into routine care for this high-risk population. Future research should focus on evaluating the effectiveness of multidisciplinary approaches and tailored interventions to reduce both the clinical and economic burden associated with comorbid depression and hypertension. Policymakers and healthcare providers should consider developing comprehensive management strategies that address both physical and mental health needs in older adult.

## Data Availability

The original contributions presented in the study are included in the article/Supplementary material, further inquiries can be directed to the corresponding author.
