# Supplementary material for: The impact of depression on mortality among older adult patients with hypertension: a systematic review and meta-analysis
Source: Front Public Health. 2025 Jul 31;13:1603785. doi: 10.3389/fpubh.2025.1603785 (PMC12350467; doi:10.3389/fpubh.2025.1603785)
Supplement: Supplementary file 1 [file Table_1.docx]

Supplementary Text 1

| Quality In Prognosis Studies tool | | | | | | | |
| --- | --- | --- | --- | --- | --- | --- | --- |
| **Author/year** | **Study Participation** | **Study Attrition** | **Prognostic Factor Measurement** | **Outcome Measurement** | **Study Confounding** | **Statistical Analysis and Reporting** | **Overall Risk of Bias** |
| Junfeng Zhou 2024 | Low risk | Low risk | Low risk | Low risk | Moderate risk | Low risk | Moderate risk of bias |
| Ruth Peters 2010 | Low risk | Low risk | Low risk | Low risk | Moderate risk | Low risk | Moderate risk of bias |
| Osvaldo P. Almeida 2019 | Low risk | Low risk | Low risk | Low risk | Moderate risk | Low risk | Moderate risk of bias |
| Jinghua Yin 2022 | Moderate risk | Low risk | Low risk | Low risk | Low risk | Low risk | Moderate risk of bias |
| Shaochen Guan 2021 | Moderate risk | Moderate risk | Low risk | Low risk | Low risk | Low risk | Moderate risk of bias |
| Xunjie Cheng 2022 | Low risk | Low risk | Low risk | Low risk | Low risk | Low risk | Low risk of bias |
| Nicholas Graham 2019 | Low risk | Low risk | Low risk | Low risk | Moderate risk | Low risk | Moderate risk of bias |
| Cyrus SH Ho 2016 | Moderate risk | Low risk | Low risk | Low risk | Moderate risk | Low risk | Moderate risk of bias |
| Lan Zhu 2024 | Moderate risk | Low risk | Low risk | Moderate risk | Low risk | Low risk | Moderate risk of bias |
| Wa Cai, MD 2023 | Low risk | Moderate risk | Moderate risk | Low risk | Moderate risk | Low risk | High risk of bias |
| R. Neal Axon 2010 | Moderate risk | Low risk | Moderate risk | Low risk | Low risk | Low risk | Moderate risk of bias |
| Josephine M.L. de Hartog-Keyzer 2022 | Moderate risk | Low risk | Moderate risk | Low risk | High risk | Moderate risk | High risk of bias |
| Haibin Li 2019 | Low risk | Low risk | Low risk | Moderate risk | Low risk | Low risk | Moderate risk of bias |
