# Supplementary material for: The impact of depression on mortality among older adult patients with hypertension: a systematic review and meta-analysis
Source: Front Public Health. 2025 Jul 31;13:1603785. doi: 10.3389/fpubh.2025.1603785 (PMC12350467; doi:10.3389/fpubh.2025.1603785)
Supplement: Supplementary file 2 [file Table_2.docx]

| Grading of Recommendations, Assessment, Development, and Evaluations | | | | | | | |
| --- | --- | --- | --- | --- | --- | --- | --- |
| **Outcome Indicator** | **Study Design** | **Risk of Bias** | **Inconsistency** | **Indirectness** | **Imprecision** | **Publication Bias** | **Quality of Evidence** |
| Effect of Depression on All-Cause Mortality in Elderly Hypertensive Patients | Cohort Study + RCT | Moderate | Moderate | Low | Low-Moderate | Low | moderate |
